# Clinical challenges in diagnosing idiopathic mesenteric phlebosclerotic colitis: two case reports and an up-to-date literature review

**DOI:** 10.3389/fgstr.2025.1549662

**Published:** 2025-04-15

**Authors:** MingYan Shang, Liang Yin, Zhangzhu Li, Jie Gan, Jing Wang

**Affiliations:** Medical Imaging Center, Shandong Provincial Third Hospital, Jinan, China

**Keywords:** idiopathic mesenteric phlebosclerosis, mesenteric vein calcification, case report, drug-related, rare gastrointestinal disorders

## Abstract

Idiopathic mesenteric phlebosclerotic colitis (IMP) is a rare ischemic colitis primarily affecting the right colon, characterized by mesenteric venous sclerosis and calcification. Its etiology remains unclear, but prolonged use of traditional Chinese herbal medicines, particularly those containing gardeniae fructus, has been suggested as one of several contributing factors, alongside metabolic disorders and potential genetic predispositions. This case report highlights two cases of IMP linked to herbal medicine use, providing insights into its pathogenesis, diagnosis, and management. Both patients were diagnosed with IMP based on CT imaging and histopathology. Management involved discontinuation of herbal treatments, dietary modifications, and controlling metabolic conditions. Both patients had stable outcomes with no disease progression upon follow-up. This case report underscores the importance of considering IMP in patients with unexplained ischemic colitis, particularly those with a history of long-term herbal medicine use. Early intervention, including discontinuation of herbal remedies and management of metabolic factors, is crucial for preventing disease progression. Further research is needed to better understand the pathophysiology of IMP and its relationship with herbal medicine.

## Introduction

Idiopathic mesenteric phlebosclerotic colitis (IMP) is a rare form of chronic ischemic colitis characterized by progressive sclerosis and calcification of mesenteric veins (1, 2), predominantly affecting the right colon (3). While its exact etiology remains uncertain, long-term use of traditional Chinese herbal medicines, particularly those containing gardeniae fructus, has been suggested as a potential contributing factor (4–6). Metabolites of gardeniae glycosides are hypothesized to induce venous endothelial damage, fibrosis, and subsequent ischemia. Additionally, metabolic disturbances and chronic inflammation may exacerbate the condition (7, 8).

The nonspecific symptoms of IMP, such as abdominal pain and altered bowel habits, pose significant diagnostic challenges. Computed tomography (CT), which often reveals mesenteric venous calcification and thickened colonic walls, plays a crucial role in diagnosis (9), while histopathological examination confirms vascular sclerosis and chronic inflammation.

In this study, we report two cases of IMP associated with prolonged herbal medicine use, emphasizing the diagnostic challenges, clinical features, and potential pathogenesis. This report aims to address the diagnostic challenges of IMP and contribute to understanding its diverse etiological factors. These cases are contextualized within a literature review to enhance the understanding and clinical recognition of this rare disease.

## Case description

### Case 1

A 48-year-old male patient with a history of chronic dermatophytosis presented with persistent lower abdominal pain lasting over 10 days. He had been undergoing long-term treatment with traditional Chinese herbal medicine and denied any changes in bowel habits, fever, or weight loss. On physical examination, tenderness was noted in the lower abdomen without rebound pain, bowel sounds were normal, and no palpable masses were detected. Laboratory tests revealed significantly elevated inflammatory markers, including high-sensitivity C-reactive protein (HS-CRP) at 43.99 mg/L (normal range: 0–3 mg/L) and C-reactive protein (CRP) at 58.8 mg/L (normal range: 0–8 mg/L), indicating systemic inflammation. A fecal occult blood test was positive, suggesting gastrointestinal bleeding.

**Figure 1:**
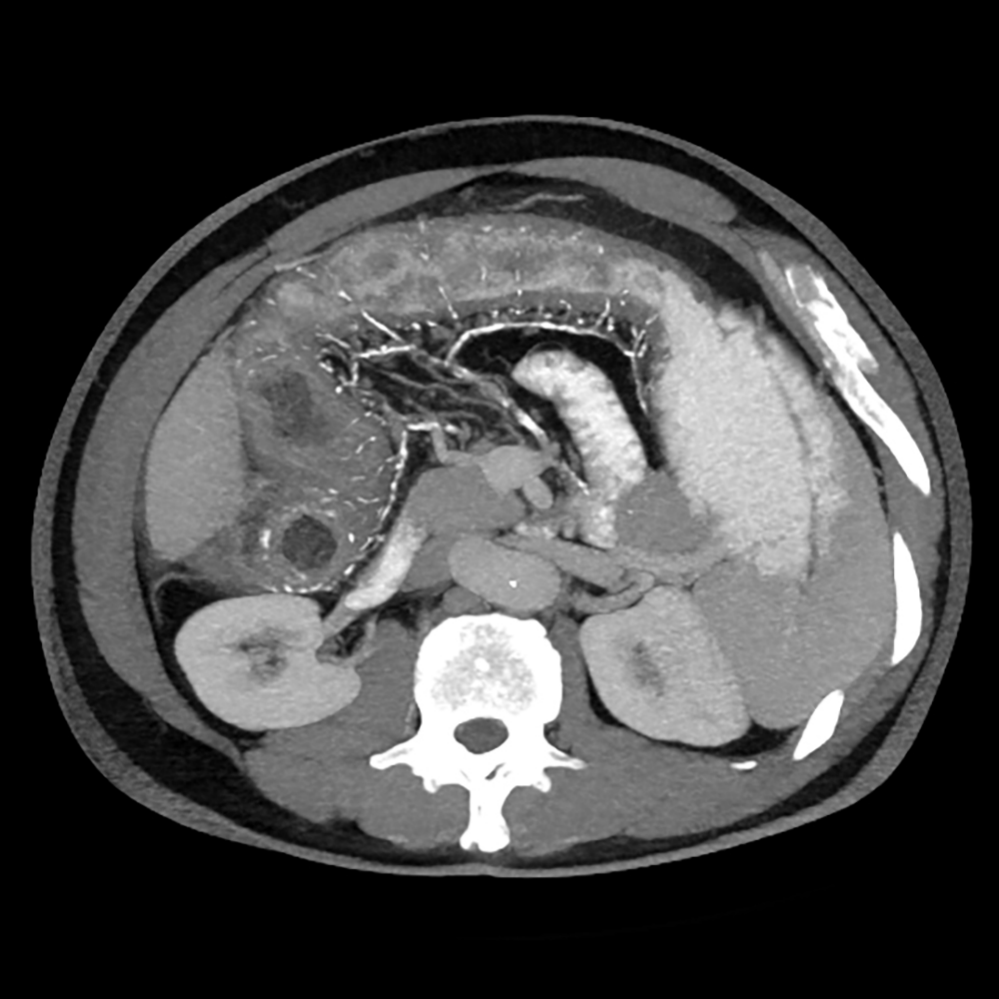
The CT image shows mild thickening of the walls of the ascending and transverse colon, with irregular margins of the ascending colon, peripheral exudation, and multiple calcifications observed in the mesenteric veins and their branches.

**Figure 2:**
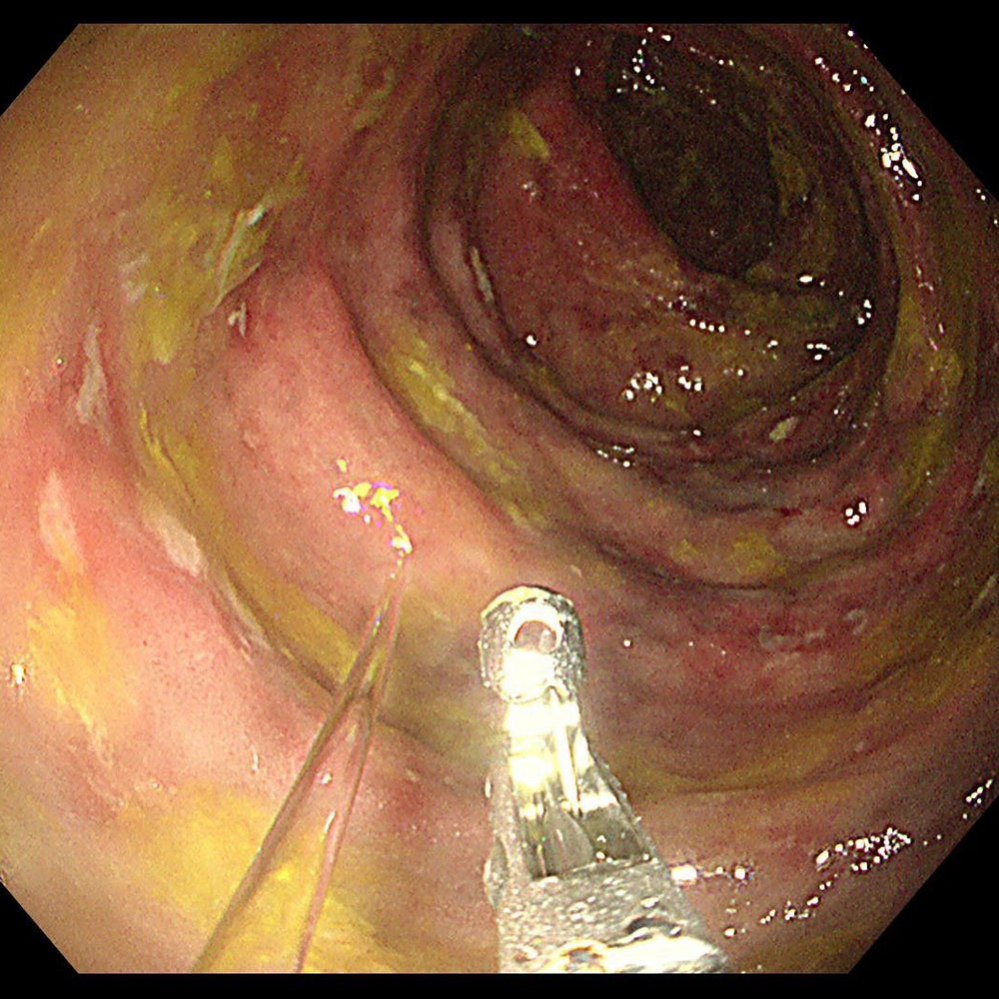
Colonoscopy showed mucosal congestion and edema with irregular small ulcers covered by white exudate. Dark purple discoloration and nodular protrusions were observed on the affected mucosa.

The potential causes of abdominal pain included intestinal inflammation, ischemia, or obstruction. The systemic inflammation suggested possible infection or autoimmune disease. The positive fecal occult blood test indicated gastrointestinal bleeding. Given the patient’s long-term use of traditional Chinese herbal medicine, an adverse drug reaction was considered a contributing factor to intestinal pathology. Additionally, mesenteric venous calcification could compromise intestinal blood flow, leading to ischemic changes. Differential diagnoses included arterial ischemic colitis, inflammatory bowel disease, and malignancy. Further imaging confirmed colonic wall thickening and mesenteric venous calcification, with MPR and MIP-enhanced CT scans improving the visualization of vascular abnormalities (**Figure 1**). Colonoscopy findings of mucosal congestion, edema, irregular small ulcers with white exudates, and dark purple nodular mucosa (**Figure 2**) further supported the diagnosis.

Based on comprehensive clinical, radiological, and endoscopic findings, the patient was diagnosed with IMP, a rare ischemic colitis associated with venous sclerosis. The treatment plan included dietary modifications to reduce intestinal burden and discontinuation of herbal medicine with potential gastrointestinal effects. A follow-up imaging assessment was scheduled for three months to evaluate changes in colonic wall thickening, mesenteric venous calcifications, and mucosal abnormalities. The results indicated no disease progression. A long-term monitoring plan was implemented to ensure disease stability and assess potential complications over time.

### Case 2

**Figure 3:**
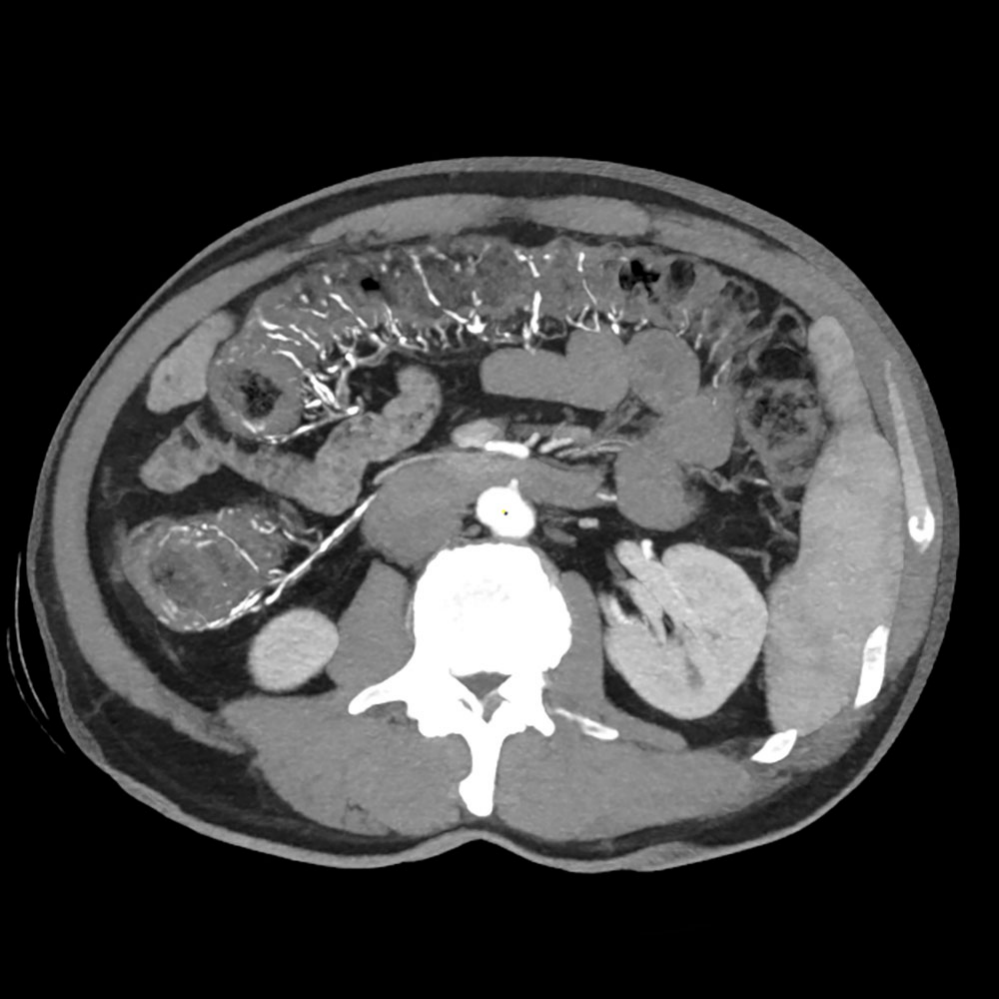
MPR, thin-layer MIP images showed slight wall thickening of the ascending and transverse colon and calcification of mesenteric veins and intestinal wall veins.

**Figure 4:**
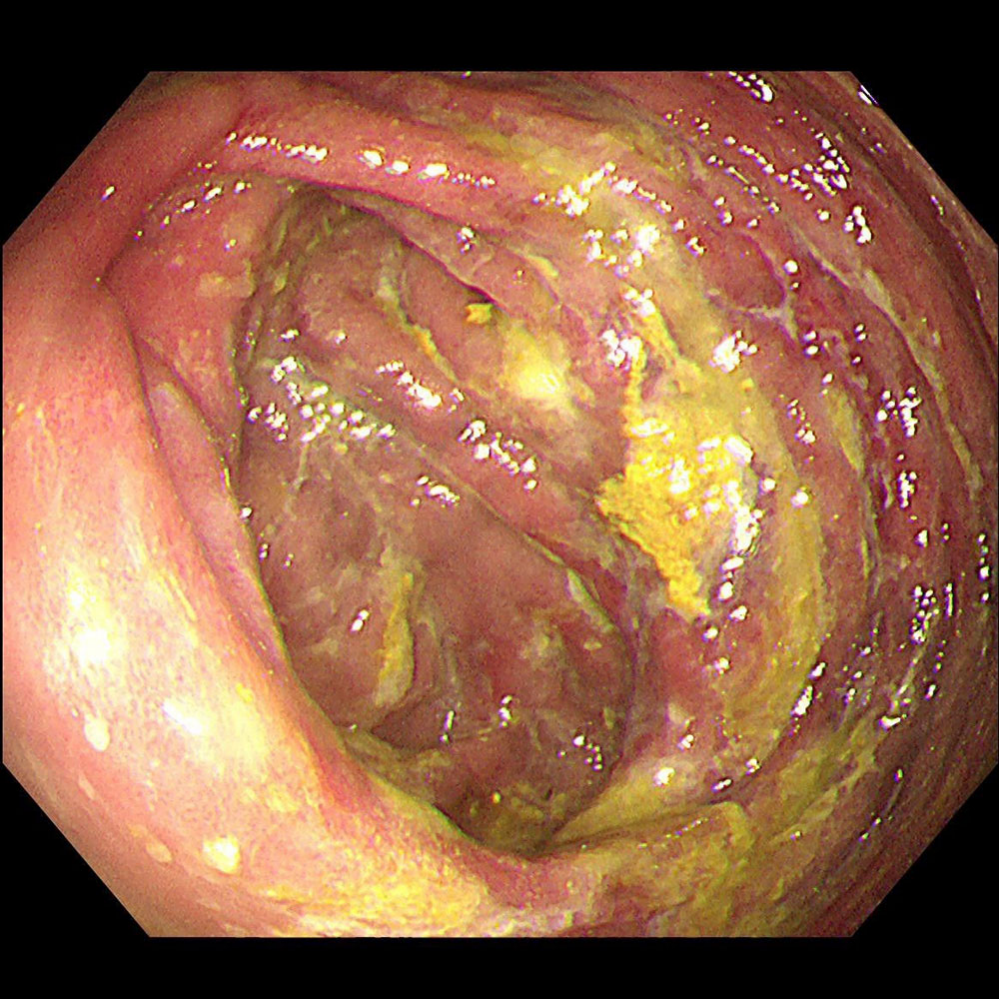
Colonoscopy showed congestion, edema and erosion of the colonic mucosa, scattered ulcers covered with white moss.

A 52-year-old male patient presented with a seven-day history of progressively worsening abdominal pain. He had a medical history of Behçet’s disease and had been undergoing long-term treatment with traditional Chinese herbal medicines, including Yinzhihuang oral liquid, for obstructive jaundice. Prior to admission, he reported intermittent fever, jaundice affecting the skin and mucous membranes, and scleral icterus. On physical examination, the patient exhibited epigastric tenderness and hepatomegaly but showed no signs of peritoneal irritation. Laboratory tests revealed hyperglycemia (8.0 mmol/L; normal range: 3.9–6.1 mmol/L), abnormal liver function, and elevated inflammatory markers. Abdominal radiography demonstrated linear calcifications within the mesenteric veins, while CT scans revealed extensive mesenteric venous calcifications and colonic wall thickening, particularly in the ascending colon, hepatic flexure, and transverse colon (**Figure 3**). Colonoscopy identified mucosal abnormalities (**Figure 4**).

A differential analysis was conducted to determine the possible causes: the abdominal pain could be attributed to intestinal pathology, hepatic dysfunction, or other intra-abdominal conditions. The jaundice, associated with liver function abnormalities, was likely due to the progression of obstructive jaundice or an adverse drug reaction. The intermittent fever suggested an underlying inflammatory response. The patient’s long-term use of herbal medicines, particularly those containing Gardenia jasminoides, was considered a potential contributing factor to both intestinal and hepatic pathology. Additionally, mesenteric venous calcification may have compromised intestinal blood supply, leading to ischemic changes. Based on radiological findings and the patient’s history of prolonged Gardenia-based herbal medicine use, a clinical diagnosis of IMP was established. Other contributing factors, such as hyperlipidemia and long-term calcium supplementation, may have further exacerbated disease severity.

The patient received supportive treatment, including symptom relief for abdominal pain, liver function improvement, inflammation control, and measures to enhance intestinal blood supply. Herbal medicine use was discontinued. During a 10-month follow-up period, the patient underwent regular symptom assessments, laboratory tests, and imaging evaluations. The results indicated a stable disease course, with no significant changes in mesenteric venous calcification or colonic wall thickening compared to initial imaging findings.

## Discussion

IMP is a rare intestinal ischemic syndrome with an insidious onset and progressive course. The term “idiopathic mesenteric phlebosclerosis” was introduced by Iwashita et al (10) in 2003 to differentiate the condition from ischemic colitis associated with arterial diseases, as the affected areas in IMP display minimal inflammatory changes. IMP is typically attributed to chronic ischemia of the colon caused by mesenteric venous calcification, which results in venous congestion and, in severe cases, hemorrhagic infarction (11). Due to its nonspecific clinical manifestations and potential for serious complications, accurate diagnosis and effective management are critical. The cases presented in this study offer valuable insights into the multifactorial nature of IMP and underscore the importance of integrating clinical, radiological, and histopathological evidence to improve recognition of this condition.

IMP remains challenging due to its nonspecific symptoms, such as abdominal pain and changes in bowel habits (12, 13). These clinical features often overlap with other gastrointestinal disorders, leading to delayed diagnosis. A review of the literature reveals that IMP can be associated with intestinal stenosis, necrosis, and obstruction (3, 14, 15), and it carries a potential risk of malignant transformation (13, 16, 17). The clinical presentation is typically atypical and nonspecific. Familial occurrences of IMP have been reported, with cases documented in spouses or between mothers and their children (8, 18), suggesting a strong correlation with the concurrent use of traditional Chinese herbal medicines within families. Therefore, we support the theory that traditional Chinese medicine likely plays a significant role in the pathogenesis of IMP. However, whether self-healing herbs or other specific herbal compounds directly contribute to IMP development warrants further investigation through studies involving larger datasets.

While emphasizing the role of Gardenia jasminoides and its active compound geniposide in the pathogenesis of IMP, other factors such as lifestyle habits, metabolic disorders, and systemic diseases may also contribute to disease progression (19). Metabolic comorbidities, including diabetes, hyperlipidemia, and chronic renal dysfunction, can exacerbate vascular remodeling and calcification. Additionally, in Case 2 of our report, the coexistence of IMP with Behçet’s disease suggests that the pathophysiology of IMP may extend beyond localized venous sclerosis and be closely associated with systemic inflammatory states. Furthermore, excessive intake of vitamin D and calcium supplementation may have played a role in promoting vascular calcification, potentially accelerating the progression of IMP (20).

The majority of IMP cases have been reported in East Asian countries, particularly Japan, the mainland and Taiwan of China, while cases outside of Asia remain rare. This geographic distribution suggests that regional factors—such as dietary habits, environmental exposures, or the widespread use of traditional herbal medicines containing gardeniae fructus—may contribute to the pathogenesis of the disease. Previous studies indicate that the average age of onset for IMP ranges from 62 to 75 years. While most studies report a slight female predominance (21, 22), the existing case reports may be insufficient to conclusively determine a gender-specific difference in the incidence of IMP (7).

IMP presents with nonspecific clinical manifestations and laboratory findings, making clinical diagnosis largely dependent on imaging and endoscopic evaluations. The diagnostic criteria for IMP are as follows (23, 24): (1) Abdominal X-ray showing multiple fine, linear calcifications in the right abdomen, oriented perpendicular to the long axis of the colon; (2) CT scan revealing multiple small, slender, and tortuous calcifications in the mesenteric veins accompanied by colonic wall thickening; (3) Air-barium enema demonstrating luminal narrowing of the right colon and the presence of a ‘thumbprinting’ sign; (4) Endoscopy showing dark purple discoloration of the mucosa, accompanied by edema, erythema, erosion, and ulceration. Therefore, in patients with typical imaging and endoscopic features, a combination of two diagnostic modalities is sufficient for diagnosis (25). In the cases described, CT imaging played a key role in identifying hallmark features, such as linear calcifications along the mesenteric veins and thickening of the colonic walls. Advanced imaging modalities, including MPR and MIP images, further enhanced the visualization of vascular abnormalities, facilitating more precise diagnosis.

Histopathological findings in IMP lack specific characteristics (26), primarily displaying mucosal active or chronic inflammatory cell infiltration, without evidence of venous thrombosis, amyloid deposition in the venous walls, or inflammatory cell infiltration. Chang KM identified unique coagulative necrosis in the veins of IMP patients (11). Research on IMP-related biomarkers using immunofluorescence (IF) and immunohistochemistry (IHC) remains limited (11), and their precise role in disease diagnosis has yet to be clearly established. Future studies should further explore the potential diagnostic and therapeutic implications of these biomarkers to enhance the understanding and management of IMP.

The management of IMP primarily depends on the severity of the disease. Mild cases are typically managed conservatively, involving the cessation of herbal medicine use and the implementation of supportive measures such as dietary modifications and symptomatic treatment (27). It remains unclear whether patients require follow-up after discontinuing herbal medications or the optimal duration of such follow-up; However, existing reports indicate that surgical intervention is generally not required for most patients after discontinuation of herbal treatments (21). Notably, Sze SF reported a single case in which a patient developed intestinal obstruction and recurrent vomiting three years after stopping herbal medication, ultimately necessitating a right hemicolectomy (28). Addressing underlying metabolic abnormalities is crucial to preventing further disease progression. In the cases discussed, patients responded well to conservative management, highlighting the importance of early intervention. Conversely, advanced cases presenting with complications such as bowel obstruction, perforation, or hemorrhagic infarction may necessitate surgical intervention (1). Subtotal colectomy or segmental resection are common surgical approaches (25), and most patients achieve favorable postoperative outcomes.

Close monitoring and follow-up are essential for all IMP patients to detect potential disease recurrence or complications. While the association between IMP and malignancy remains unconfirmed, further research is needed to evaluate long-term outcomes, including the potential for malignant transformation (29).

The cases presented in this study highlight several critical considerations for clinicians. IMP should be included in the differential diagnosis of unexplained ischemic colitis, particularly in patients with a history of herbal medicine use or regional risk factors (25). The combination of advanced imaging techniques and detailed clinical histories is crucial for accurate diagnosis (30). Although the potential biomarkers for IMP have not yet been clearly identified, inflammatory markers, circulating endothelial injury markers, and metabolites associated with gardeniae fructus exposure may serve as possible candidates. Early detection and monitoring using these indicators could significantly enhance diagnostic accuracy and therapeutic outcomes (11, 31). Additionally, educating patients on the potential risks associated with herbal medicine and the importance of managing metabolic comorbidities can contribute to disease prevention and effective management.

Future research directions include conducting larger multicenter studies to better understand the pathophysiology and risk factors of IMP. This involves applying proteomics and metabolomics techniques to screen for potential biomarkers, as well as exploring the dose-response relationship between geniposide exposure and venous sclerosis. Multicenter prospective observations should be conducted to evaluate venous sclerosis and to construct dose-response models. These studies will provide deeper insights into the underlying mechanisms and support the development of targeted therapies.

## Conclusion

IMP is a complex, multifactorial disease that should be considered in clinical practice, particularly in patients presenting with unexplained ischemic colitis and a history of herbal medicine use. The presence of multiple tortuous calcifications within the mesenteric veins, accompanied by colonic wall thickening, represents a characteristic imaging feature that, when combined with a detailed clinical history, can significantly enhance diagnostic accuracy. Further research is needed to elucidate the specific risk factors and underlying etiology of IMP, with the goal of reducing its incidence and identifying high-risk populations. The cases presented in this study underscore the importance of clinician awareness, patient education, and ongoing investigation into the pathogenesis and treatment of this rare condition.

## Data Availability

The raw data supporting the conclusions of this article will be made available by the authors, without undue reservation.
